# MedicalCLIP: Anomaly-Detection Domain Generalization with Asymmetric Constraints

**DOI:** 10.3390/biom14050590

**Published:** 2024-05-16

**Authors:** Liujie Hua, Yueyi Luo, Qianqian Qi, Jun Long

**Affiliations:** 1 School of Computer Science and Engineering, Central South University, Changsha 410083, China; liujiehua@csu.edu.cn; 2 School of Mathematics and Statistics, Central South University, Changsha 410083, China; luoyueyi@csu.edu.cn; 3 Big Data Institute, Central South University, Changsha 410083, China; qiqianqian@csu.edu.cn

**Keywords:** anomaly detection, multimodal contrastive learning, domain generalization, GPT

## Abstract

Medical data have unique specificity and professionalism, requiring substantial domain expertise for their annotation. Precise data annotation is essential for anomaly-detection tasks, making the training process complex. Domain generalization (DG) is an important approach to enhancing medical image anomaly detection (AD). This paper introduces a novel multimodal anomaly-detection framework called MedicalCLIP. MedicalCLIP utilizes multimodal data in anomaly-detection tasks and establishes irregular constraints within modalities for images and text. The key to MedicalCLIP lies in learning intramodal detailed representations, which are combined with text semantic-guided cross-modal contrastive learning, allowing the model to focus on semantic information while capturing more detailed information, thus achieving more fine-grained anomaly detection. MedicalCLIP relies on GPT prompts to generate text, reducing the demand for professional descriptions of medical data. Text construction for medical data helps to improve the generalization ability of multimodal models for anomaly-detection tasks. Additionally, during the text–image contrast-enhancement process, the model’s ability to select and extract information from image data is improved. Through hierarchical contrastive loss, fine-grained representations are achieved in the image-representation process. MedicalCLIP has been validated on various medical datasets, showing commendable domain generalization performance in medical-data anomaly detection. Improvements were observed in both anomaly classification and segmentation metrics. In the anomaly classification (AC) task involving brain data, the method demonstrated a 2.81 enhancement in performance over the best existing approach.

## 1. Introduction

Anomaly detection is widely applied across various sectors including industrial production [1,2,3], finance, autonomous driving [4], and disease diagnosis [5,6,7,8,9,10]. In the medical field, anomaly detection can help reduce misdiagnoses and missed diagnoses caused by human error during manual inspections. Compared to the industrial sector, medical data requires a higher degree of specialization. The rarity and diversity of anomaly data make model construction in this context particularly challenging. Traditional methods, which rely on the completeness and availability of data [11,12], often struggle in this context. Constraints related to data privacy and the scarcity of anomaly data further complicate the direct training of anomaly-detection models.

Existing anomaly-detection methods typically train specifically on certain datasets, requiring the construction of multiple models and extensive training to adapt to different application scenarios [13]. This limits the performance of supervised methods and has become a significant bottleneck in medical-data anomaly detection [14,15,16,17]. Additionally, in multi-category anomaly-detection tasks, multiple models necessitate substantial computational power and storage resources. Accordingly, the pursuit of a unified generalization model for anomaly detection, applicable across diverse data categories, has become a pivotal area of research. Domain-generalization methods offer new solutions to tackle these challenges, enabling models to perform effectively across various unknown environments by leveraging generalized features that are not tied to the specifics of any single dataset.

Domain generalization [5,18,19,20] aims to build models for data from unknown domains, which addresses challenges such as data scarcity and inaccessibility in new fields. Given the limitations of available data and the diversity of anomalies, enhancing the domain generalization of models for such tasks is crucial. However, current research in image-based anomaly detection tends to focus excessively on single-modal, domain-specific data, neglecting broader, domain-independent representations. Traditional anomaly detection typically involves only image modality data. Relying exclusively on image data’s feature distribution for constructing classification representations, single-modal approaches significantly limit the model’s ability to generalize across different domains [21]. Various methods such as distribution-based representation [11], distance optimization [22,23], and adversarial generation [24,25,26] are employed. However, domain-generalization representations based on single-modal data lack diversity.

Single-type, single-modal anomaly detection relies heavily on the construction of classifiers and the distribution of normal samples, leading to models that are significantly tailored to specific datasets. Significant variations in data distributions across different categories present substantial challenges for domain generalization in models reliant on single-modal data. The development of multimodal contrastive models offers a new research direction for domain generalization in the medical-data anomaly-detection field [14]. Multimodal approaches leverage the strengths of multiple types of data inputs, such as combining image and text data, to enhance the robustness and generalization of anomaly-detection systems across different domains. This integration not only expands the representational diversity but also improves the adaptability of models to new, unseen datasets, overcoming the limitations associated with traditional, single-modal anomaly-detection methods [27].

Medical anomaly detection is a complex and resource-intensive task. It relies heavily on professionals to meticulously annotate features in datasets, a critical step that ensures accuracy during the training process [28]. Establishing orthogonal domain spaces and distributions helps to create clear representational boundaries, which are essential for effective anomaly classification. However, while the construction of medical image anomaly-detection datasets has demonstrated improved model performance due to its orthogonality, this characteristic also poses challenges to the model’s generalization ability [29]. Moreover, the distribution of source-data representations is closely related to the representation space of specific domains. Establishing orthogonal domain spaces and distributions helps to create clear representational boundaries, which are essential for effective anomaly classification.

Traditional anomaly-detection models, including self-supervised [6,22,30] and generative models [25], are capable of learning image-representation distributions and normal representations from extensive medical image datasets. However, changes in the detection data lead to corresponding shifts in the distributions of data presentations and normal representations. Relying solely on single-modal data representation makes it difficult to achieve a rich information representation for domain generalization and to distinguish between normal and anomalous conditions effectively.

Therefore, anomaly-detection methods enhance model domain-generalization capabilities by incorporating multimodal approaches. The integrated constraints between multimodal data enhance the model’s ability to represent information from the data [31,32,33]. Image–text models enrich the model’s capability for information representation by applying hierarchical representational constraints and building a more diversified information representation [34,35]. The inclusion of linguistic information allows a single description to correspond to multiple objects and categories, as shown in Table 1. For example, the representation of holes is more dispersed and abstract than image representations. The constraints it constructs are more broadly applicable, enhancing the model’s ability to relax the representation of broad-domain data. Supervised methods often build orthogonal feature encoding, but this approach is not conducive to domain generalization [13]. Different objects have different characteristic representations and should be measured using different orthogonal representations. In the context of unsupervised feature encoding, the resulting vectors deviate from orthogonality, with distinct objects assuming unique positions that more accurately reflect real-world conditions. The advantage of using natural language for supervision is that it allows for more diverse expansions. Linking language representations with image representations allows for more flexible transformations.

The purpose of anomaly detection is to identify data that does not conform to the normal distribution [36,37]. Due to the scarcity of anomalous data, a common approach is to distinguish anomalies by learning only the feature distribution of normal data [11,22]. This requires the extracted data representations to be highly orthogonal, and the model is constructed using a single type of data. Image generation methods generate images of the normal distribution for specific categories of data and reconstruct feature distributions [25]. Common detection methods include classification-based [2,7] and generation-based methods [38]. Given the scarcity of anomalous data, learning the feature distribution of normal data is key to effective detection. Data representations are typically orthogonal and tailored to specific data categories [1,22]. On the other hand, image generation methods focus on reconstructing the distribution of normal samples for specific categories.

Domain-generalization techniques are employed to enhance anomaly detection in image-based texture and surface defect identification [31]. Large-language pre-trained models (such as VLP [39], ALIGN [40]) demonstrate great adaptability in feature consistency expression and model generalization capabilities, achieving cross-modal information interaction through global information expression and similarity comparison. Multimodal large models have shown good results in anomaly detection through contrastive learning [41], generative learning, and large model denoising methods. The multimodal invariant representation anomaly-detection method improves model generalization performance by learning domain-invariant representations [42]. For more granular detection, image patch and meta-learning-based methods are applied in anomaly detection.

**Figure 1:**
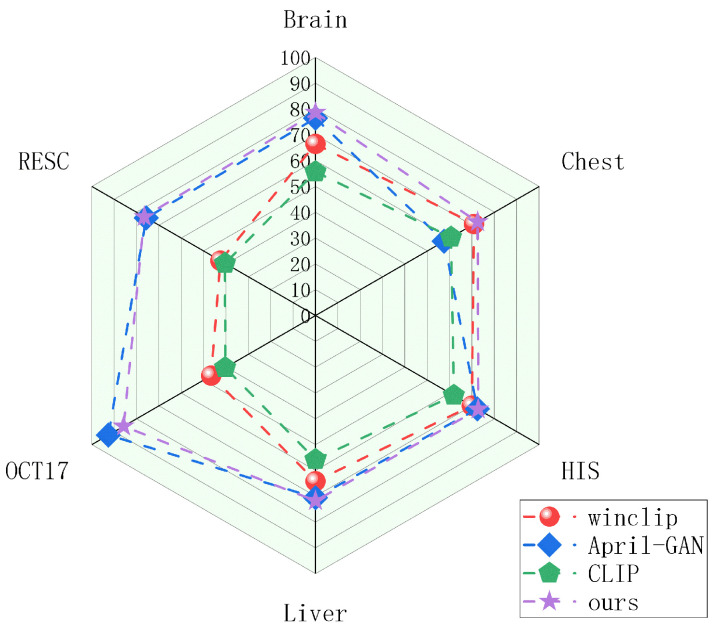
Domain-generalization model for zero-sample anomaly classification method for different data. Compared to the existing methods, our method shows competitive results.

Based on the issues discussed, MedicalCLIP explores a unified medical anomaly-detection model with strong generalization capabilities. By utilizing spatially consistent representations of multimodal data, the model achieves not only unification but also an enhancement of its generalization capabilities. Through image–text comparative learning, a category-independent model for domain generalization in anomaly detection is implemented [43]. Constructing a unified anomaly-detection model with robust domain-generalization capabilities is of significant practical value for improving model efficiency and achieving model generalization. Figure 1 demonstrates the classification capabilities of MedicalCLIP.

For anomaly-detection tasks, through the comprehensive comparative representations within and between modalities, we find that the intramodal representation constraints of different modal data have varying impacts. Common anomaly-detection methods involve contrastive learning among normal samples, but they often overlook the model limitations caused by spatial differences. A unified anomaly-detection model should be able to extract more applicable representational data, utilizing multimodal data integration and multitask loss for adaptive feature extraction. In the processing of single-image modal data, models primarily focus on the visual representation of the image, lacking guidance from other modal data, making it difficult to extract logical information from the image. Introducing textual descriptions, such as “This is a bottle with cracks”, enables the accurate identification and extraction of key elements in the image, such as the number of bottles and defects, by leveraging semantic information. Multimodal integrated contrastive encoding, when compared to image data, enhances the textual representation’s guidance on image representation. MedicalCLIP explores a medical-data anomaly-detection method with strong generalization capabilities that uses multi-angle, cross-modal reasoning. This prevents any single modality from dominating the entire model-training process, therefore learning more universal information representations and enhancing the associative capabilities between different modal data.

Movtivation: For medical-data anomaly-detection tasks, this paper proposes an asymmetric constraint MedicalCLIP domain-generalization method for anomaly detection and segmentation. By implementing intramodal constraints within image and text modalities, the consistency of image modality representations and the constraints within and between modalities are enhanced, thus balancing the model’s domain generalization and detection effectiveness.

## 2. Materials and Methods

The purpose of MedicalCLIP is to train a unified model capable of achieving anomaly detection through zero-shot learning. For the given training data $D_{train}=\left\{I_{train},Y_{train}\right\}$, the images $x^{I}\in I_{train}$ and label of images $Y_{train}\in \left\{0,1\right\}$. The test dataset $D_{test}=\left\{D_{test}^{1},D_{test}^{2},\cdots ,D_{test}^{m}\right\}$, *m* is the classes of test dataset, $D_{train}\cap D_{test}=⌀$. The model is trained by the given dataset Dtrain, resulting in superior anomaly-detection performance on the test set Dtest. There are information differences between different categories of data, i.e., $D_{test}^{1},D_{test}^{2}$, making it difficult to distinguish between different types of data in the feature space. We employ a multimodal approach to develop a unified model capable of handling multiclass anomaly detection. For the given image, we introduce textual T to guide the representation of the image data and conduct multimodal contrastive analysis based on textual information. The calculation method for anomalies is to compute the similarity between data of different modalities. We characterize the data using the image encoder $f_{\phi }\left(x^{I}\right)$ and the text encoder $g_{\theta }\left(x^{T}\right)$. The intermodal contrast losses are as follows:

(1) $$L\left(I,T\right)=min<f_{\phi }\left(x_{i}^{I}\right),g_{\theta }\left(x_{j}^{T}\right)>$$

where $L_{CL}$ denotes the contrast loss, $f_{\phi }\left(x_{i}^{I}\right)$ and $g_{\theta }\left(x_{j}^{I}\right)$ denotes text and images of the same category, and $f_{\phi }\left(x_{i}^{I}\right)$, $g_{\theta }\left(x_{j}^{I}\right)$ denotes text and images of different categories.

(2) $$L_{cl,I\to T}=\frac{-1}{n}\sum_{i=1}^{n}\log\frac{\exp(\langle f_{\phi }\left(x_{i}^{I}\right),g_{\theta }\left(x_{i}^{T}\right)/\tau )}{\sum_{j\in [n]}\exp(\langle f_{\phi }\left(x_{i}^{I}\right),g_{\theta }\left(x_{j}^{T}\right)/\tau )}$$

The purpose of MedicalCLIP is to establish fine-grained constraints within modalities for comprehensive representation and to achieve more fine-grained information filtering using comprehensive constraints L between image-image $f_{\phi }\left(x^{I}\right)$ and text-text $g_{\theta }\left(x^{T}\right)$. The CLIP model possesses powerful feature extraction capabilities by comparing text and image contrast representations. MedicalCLIP optimizes the fine-tuning of the training data through contrast embedding between image modalities and textual modalities and guides the image learning anomaly-detection data representation by adaptively generating textual cues.

### 2.1. Overview of Framework

**Figure 2:**
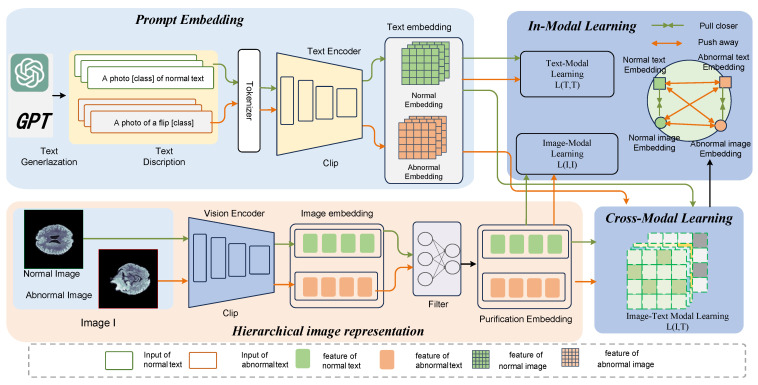
Overall framework of MedicalCLIP. The prompt embedding section includes prompt generation and text feature extraction. The hierarchical image-representation section preserves features at different levels of the image feature extraction representation and refines the image representation through a filtering module. The asymmetric constraint module contains cross-modal constraints and modal content.

**Figure 3:**
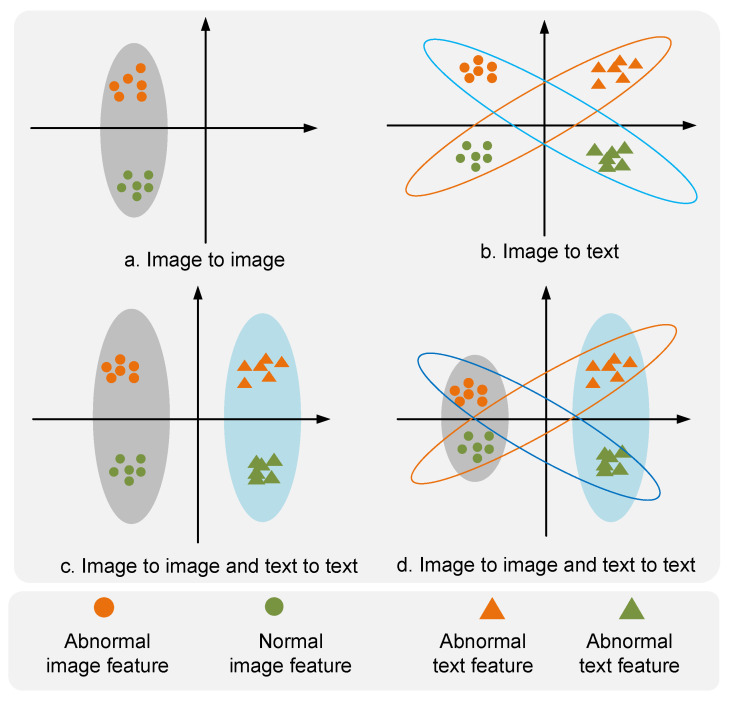
Irregular constraints. (**a**) represents the constraints for image category data. Crossing constraints between graphics are represented in (**b**). (**c**) represents constraints between image and image, text and text. (**d**) represents the multimodal irregular constraint method. The blue and orange circles indicate the sample constraints for different modes, respectively **cad: An [image] photo of a [state].**

As illustrated in Figure 2, the framework of MedicalCLIP is structured into four primary components: 1. Promote Embedding. GPT [44] is utilized to generate textual corpora T, and leveraging the Contrastive Language-Image Pre-training (CLIP) [45] template, we craft both standard and anomalous textual descriptions tailored for diverse image categories. 2. Hierarchical image representation Constructing an image–text corpus $X={\left\{x_{i}^{T},x_{i}^{I}\right\}}_{i=1}^{n}$ from the generated text, we construct comprehensive comparison methods based on the same modality and different modalities. 3. In-Modal Learning By integrating an asymmetric image–text constraint L, we bolster the synergy between modalities, ensuring the model offers a harmonized representation of anomalous data. Image T and textual I intermodal comparison module, which deepens cross-modal understanding by comparing feature differences between text and images; Text and image intramodal comparison module, which focuses on feature comparisons within their respective modalities to improve the model’s detailed representation of the data, as is shown in Figure 3.

Multimodal comparative learning The vision–language model (VLM), which aims to maximize consistency between $x^{T}$ and $x^{I}$, has a limited ability to characterize details. For AD tasks, anomalies are often not obvious, and therefore, more detailed image representations need to be extracted. Relying on intermodal contrast constraints alone is not sufficiently capable of characterizing the lower-order information of an image. We propose a novel irregular multimodal constraint(IRC) technique to improve the model’s understanding of the distribution of image and text modalities through intramodal data constraints.

(3) $$L_{IRC}=<f_{\phi }(x_{i}^{I},f_{\phi }\left(x_{j}^{I}\right)>-\lambda <f_{\phi }(x_{i}^{T},f_{\phi }\left(x_{j}^{T}\right)>,$$

λ is the asymmetry factor. The representation of multimodal semantic information can bolster the invariance of features during the domain-generalization process. Within multimodal information, the capability of text $f_{\phi }\left(x^{I}\right)$ to represent data information is enhanced through adaptive text generation. Given the limited capacity of intermodal contrastive representations for detailed information, we employ irregular constraints to improve the model’s ability to represent data across different modalities. Moreover, in the process of anomaly segmentation, models constrained by local intermodal interactions can focus more on local detail information. For these irregular constraints, we consider two types of comprehensive constraints across different modalities.

### 2.2. Promote Embedding

In multimodal contrastive models, textual information T serves as an anchoring guide for image representation I. We achieve a comprehensive representation of images $f_{\phi }\left(x^{I}\right)$ through adaptive generation, facilitating a more thorough information portrayal. During the text-generation process, we construct two textual generation strategies: specific image descriptions spd and category-agnostic descriptions cad. For specific image descriptions, text is generated based on the acquired image category information **[cls]**, containing more detailed image-specific details. In contrast, category-agnostic representations lean towards general textual descriptions unrelated to specific categories. Using text as an anchor point for contrastive optimization allows for the acquisition of more generalized, domain-invariant information representations.

(4) T=T(spd)+T(cad)

Prompt information generation The foundational corpus of the CLIP model was specifically designed for classification tasks, encompassing both the template $M=m_{1},\ldots ,m_{n}$ and the state $D=d1,d2,\ldots ,d_{l}$. Recognizing the multifaceted nature of anomalies, we aimed to curate a corpus tailored for anomaly detection. For a given image $x_{i}$, we first obtain the category information of the image and combine it with the template information for image description generation. Subsequently, leveraging an automated prompt generation strategy, we sculpt a corpus apt for anomaly detection. The crafted template for anomaly-detection resonates with the structure “A photo of a state object”, exemplified by “A photo of a healthy brain”. For the descriptor ensemble D=d1,…di, the embedding is achieved via the prompt template. The illustration delineates both the normal and anomalous data, showcasing their respective textual representations.

**spd: A [domain] photo of a [state] [class].**

For anomaly detection in medical imaging, each characterization is multifaceted, with each facet boasting an array of templates and descriptor terms *D*, such as “normal”, “enhanced contrast”, and “intact structure” for typical annotations. In the context of atypical scans, descriptors might encompass phrases like “presence of lesions” or “indications of calcifications”. Harnessing the robust text-generation prowess of GPT, we are equipped to craft intricate textual categorizations for distinct diagnostic categories.

(5) $$x^{T}=\sum_{i=1}^{N}FillTemplate\left\{m_{i},d_{i}\right\}$$

Merging templates with descriptor terms empowers us to formulate textual portrayals for images. When assimilating unknown data, pinpointing the image’s category and integrating it into the query template suffices for automated text generation. In juxtaposition with handcrafted corpora, this automated narrative is notably more exhaustive and intricate, particularly for datasets demanding niche expertise.

(6) $$f_{normal},f_{abnormal}=textencoder\left(prompt_{\mathrm{normal}}^{n},prompt_{\mathrm{abnormal}}^{m}\right)$$

**Figure 4:**
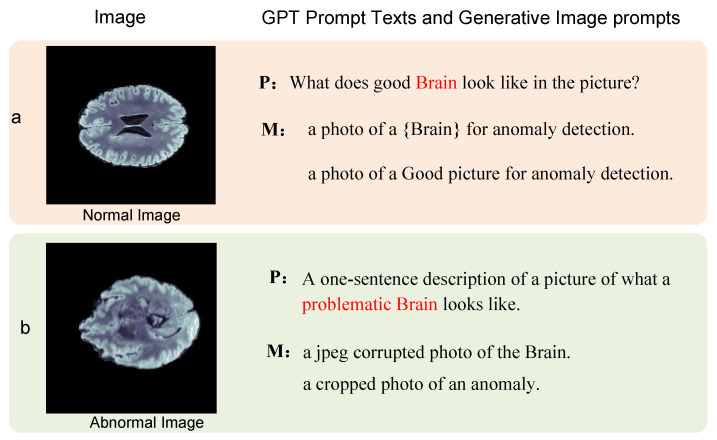
Image Text Generation. Leveraging textual cues to generate textual representations that conform to the template. (**a**) is a textual description of a normal image; (**b**) is a textual description of an abnormal image. **P** is the prompt message, and **M** denotes the text generated according to the template form.

For the given text feature representation, it consists of a combination of normal and anomalous representations. An example of the generated text representation is shown in Figure 4.

(7) $$f_{\phi }x^{I}=\left\{f_{normal},f_{abnormal}\right\}$$

(8) T=Generate{GPT(class,model)}

### 2.3. Hierarchical Feature Representation

In our method, image anomaly classification is achieved through global representations, while anomaly segmentation is accomplished using local feature representations. By employing multi-scale image feature representations, more fine-grained feature extraction is achieved.

(9) $$f_{\phi }x^{I},f_{\phi }x^{\mathrm{IP}}=imageencoder\left\{image,patch\left(image\right)\right\}$$

### 2.4. Image Feature Adaptation

The CLIP model, as a visual-language model, is primarily designed for classification tasks. Classification models, through training, make data of different categories cluster in the feature space, displaying clear, orthogonal boundaries. However, this method of data representation fundamentally differs from what is required in anomaly-detection tasks. In the context of anomaly detection, anomalies are typically sparser compared to normal data, leading to blurred boundaries and potentially non-orthogonal representations in the feature space. Confronting this challenge, we present a feature adapter, $E_{\psi }$, aimed at adjusting and aligning text and image features so that they can better adapt to the needs of anomaly-detection tasks.

(10) $$o^{T},o^{I}=E_{\psi }\{\left(g_{\theta }\left(x^{T}\right),f_{\phi }\left(x^{I}\right)\right\}$$

In the field of anomaly detection, the problem of domain generalization focuses on detecting and locating anomalies within normal images and generalizing them to untrained target domains. This primarily addresses the challenge of limited training data during the production process. For given source domain data $I^{sl}={\left\{i_{m}^{sl},y_{m}^{sl}\right\}}_{m=1}^{N_{sl}}$, where $i_{m}\in I$, y∈{0,1}, and assuming all training data are normal, the objective is to train on this normal data to achieve anomaly detection, and then apply this detection capability to data in untrained target domains. The source domain data includes image data *I* and the generated textual data *T*. The aim is to use normal image data in conjunction with adaptively generated textual data. By contrasting the representations of text and image data, a comprehensive representation and analysis of the source domain data is achieved. Within the source domain, the generation of textual data adaptively incorporates domain expert knowledge, resulting in well-characterized textual data. By contrasting data intra-modally and inter-modally, detection efficacy is enhanced. During the domain-generalization process, bridging the gap between the representations of normal and anomalous data and enhancing adaptability to the source domain data is crucial.

The vision–language model demonstrates good performance in cross-modal contrastive learning. For the given image encoder $f_{i}$ and text encoder $f_{t}$, given an image $x_{I}\in I$ and text data $x_{T}\in T$, the encoders $f_{\phi }\left(x^{I}\right)$ and $g_{\theta }\left(x^{T}\right)$ represent the image and text encoders, respectively.

Asymmetric Image-Text Constraints In anomaly detection, the generated textual information is categorized into two types: normal class descriptions and anomaly class descriptions. All images are represented as vectors of these two classes, and the textual information serves as an anchor point for calculating the loss between images and textual information. The specific process is shown in Algorithm 1.
**Algorithm 1** Feature self augmentation process  1:# I Input Image  2:# T Input Text  3:# F ← Image_Encoder()  4:# T ← Feature_Extractor()  5:# A ← Adaptor()  6:# N ← Feature_Filter()  7:pretrain_init(F)  8:**for** each x in data_loader **do**  9:# Asymmetry constraint10:# extract feature representations of different modes11:    I_f = image_encoder(I)12:    T_f = text_encoder(T)13:# Loss function14:    loss_cl = cross_entropy_loss (I_f, T_f)15:    loss_IRC = cross_entropy_loss (I_f, I_f) - β cross_entorpy_loss (T_f, T_f)16:    loss = loss_(cl) +loss_(IRC)17:    F ← F.detach()18:    update(T, D)19:**end for**

Contrastive Segmentation For image-segmentation tasks, the model needs to focus more on the fine-grained details of images. To enhance the model’s attention to image details, local representations of the image are extracted and compared with textual representations. Furthermore, during the feature extraction process of images, features at different levels contain various types of image information. Therefore, this paper implements a hierarchical representation of images to achieve a diversified association between image and text. For the given anomaly-detection method, we propose an asymmetric clip constraint domain-generalization method for anomaly detection and segmentation, which is used to perform anomaly detection and segmentation. The asymmetric constraint improves the consistency of image modality representation and the constraints within and between modal representations, enhancing the model’s balance between domain generalization and detection effectiveness.

In a manner similar to the feature similarity calculation method used in CLIP, we first obtain the hierarchical representation *x* of images and the representations *t* of various types of text. Then, we compare the similarity of intra-class features for each modal data. The input image data $x^{T},x^{I}$ is first constrained by the original CLIP model method, where the original model establishes the $x^{T}⊙x^{I}$ clip constraint by constraining the data between different modalities.

(11) $$L_{C-\mathrm{clip}}=\frac{1}{N}\sum_{j=1}^{N}\sum_{i=1}^{N}{\left(\langle x_{j}^{I},x_{i}^{T}\rangle -\lambda \langle x_{k}^{I},x_{j}^{T}\rangle \right)}^{2}.$$

Constraints between different modalities tend to focus more on the representation of semantic information. In contrast, modal content comparison constraints are more about the representation of fine-grained information. As for irregular constraints within modalities, they are as follows:

(12) $$L_{I-\mathrm{clip}}=\frac{1}{N}\sum_{j=1}^{N}\sum_{i=1}^{N}{\left(\langle x_{j}^{I},x_{k}^{I}\rangle -\beta \langle x_{k}^{T},x_{j}^{T}\rangle \right)}^{2}.$$

β is the irregular factor in irregular constraints. We use the input image and enhanced image to perform intermodal contrast constraints. The intermodal constraints are enforced in the form of element-wise dot products, $x^{T},x^{I}$, and the intramodal constraints are enforced by associating various types of image representations with text representations, enhancing the model’s understanding of a single modality. Beyond the constraints between images, there are also constraints between texts represented by $L_{clip}$. Since image data tend to represent more detail-oriented features, while text data have a more nuanced understanding of higher-order information, the intermodal constraints for images and texts are looser for images and tighter for texts.

## 3. Results

Datasets To demonstrate the wide adaptability of the MedicalCLIP model in the field of anomaly detection, we conducted validations on datasets such as brain, chest, and liver, covering various areas including multiple types of data within the medical field. During the training process, the training data only contains normal data, while the testing data includes both normal data and annotated anomaly data for evaluating the model’s performance. Moreover, through extensive ablation studies and comparative experiments across datasets from different domains, we further confirmed the generalization ability of our method in anomaly detection.

**Figure 5:**
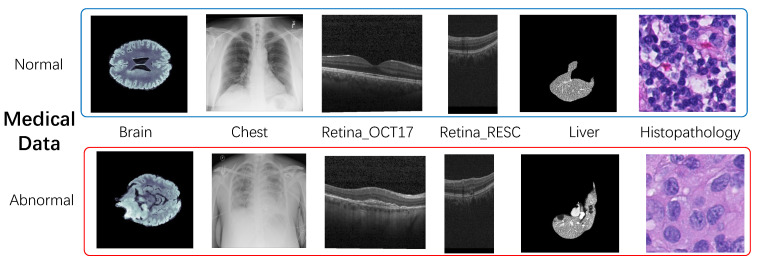
Partial samples from the Medical datasets are presented, where blue boxes indicate normal samples, while red boxes denote anomalous samples.

Metrics The performance of our model is evaluated on our medical public datasets. These datasets include brain and chest, and include normal and abnormal data, as well as annotated segmentation data. Figure 5 shows the sample image of the medical dataset. To measure the performance of the model in the process of anomaly classification (AC) and anomaly segmentation (AS), the anomaly-detection task is set to evaluate the model performance. The AUROC metrics for image anomaly detection and anomaly segmentation are used to evaluate the classification results. In anomaly detection and segmentation tasks, data categories are often imbalanced. Although anomaly-detection segmentation tasks involve pixel-level segmentation, the ultimate goal remains to distinguish between abnormal and normal areas. AUROC can evaluate the model’s ability to detect anomalies at different thresholds, and it is not affected by the imbalance in data categories.

Implementation In the image and text feature extraction models, we employ the CLIP model pre-trained by OpenAI, and the text is automatically generated by leveraging the GPT-3.5 model for template construction. The pre-training model is ViT-L/14 [46] as the MedicalCLIP backbone, and the obtained image representations and text representations are compared. CLIP’s original text features and image representations are trained for classification tasks. To align better with anomaly detection and segmentation tasks, this paper introduces an adaptive network layer for feature adaptation. The feature adaptive layer utilizes a shallow network to facilitate task adaptation for image and text representations.

In hierarchical feature representation, we use 6, 12, 18, 24 layers of representations for extraction. The software pytorch-2.1.1 used in the experiments was run using a single NVIDIA V100 32 GB GPU(The equipment was sourced from NVIDIA Corporation, located in Santa Clara, CA, USA), with an epoch setting of 50, a batch size of 16, and a learning rate of 0.0002. Throughout the model-training process, the irregular constraint β was set at 0.4 to obtain optimal results. During the training process, we adopted a multi-category unified training approach, inputting multiple different categories into the model for simultaneous training. Concurrently, while constructing a multi-category unified model, we aimed to establish an anomaly-detection model with strong domain-generalization capabilities.

### 3.1. Domain Adaptation Anomaly Detection

To evaluate the generalization ability of the model for different classes of data, we validate it on 6 different medical data. The anomaly-detection results for different categories and methods are shown in Table 2. The different models show some domain-generalization ability in medical data. Among all the methods, MedicalCLIP shows better generalization ability and superior domain-generalization performance for data such as the brain and liver.

Analysis of results Traditional anomaly-detection evaluation metrics include two primary tasks: anomaly detection and anomaly segmentation. Intramodal constraints aim to achieve a more compact feature representation within the same type of modal data. In contrast to classification models, the distributions of normal and abnormal data are largely represented within a similar data space, with only local outliers deviating from the typical representations. By constructing intramodal data contrasts between images, such as the comparison of similarity between $x^{T}$ and $x^{I}$, the focus tends to be more on semantic representation, often overlooking details. In the course of generating text, establishing a more diverse linguistic representation enriches the types of data representation.

Table 2 indicates that our method demonstrates excellent anomaly detection and segmentation performance on most datasets. However, it does not perform optimally on the OCT17 dataset. From an overall perspective, the MedicalCLIP model exhibits relatively balanced generalization capabilities across different domains, but it falls short in higher-precision detection tasks, such as on the OCT17 dataset, indicating room for improvement. For instance, on the OCT dataset, the April-GAN shows superior detection performance, yet its overall generalization ability remains limited.

**Figure 6:**
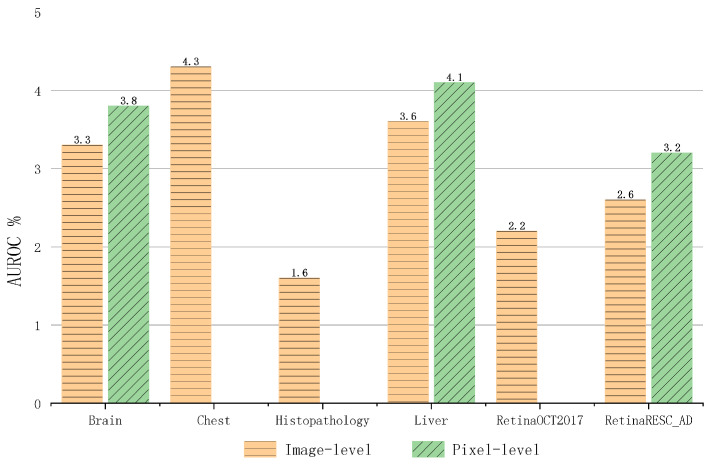
Introducing the irregular constraint, overall results demonstrate that the improvement in performance due to this constraint is more significant in segmentation tasks compared to classification tasks. The yellow color indicates an increase in the classification (AC) effect, and the green color indicates an increase in the segmentation (AS) effect.

Given the frequent changes in data distribution, anomaly detection becomes crucial for identifying distributional anomalies within normal data representations. The primary challenge lies in enhancing the model’s sensitivity to shifts in data distribution while simultaneously improving its capacity to detect such changes within similar types of data. In the process of enforcing intermodal constraints, we enhance the model’s capability for diversity transfer. Concurrently, applying intramodal constraints boosts the model’s relevance transfer. The improvement in classification and segmentation results is shown in the Figure 6.

### 3.2. Irregular Constraints

In the context of irregular constraints, different degrees of constraints have varying impacts on the effectiveness of detection. During the process of anomaly segmentation, the model pays more attention to the details of the image, thus imposing stronger constraints on the image.

**Figure 7:**
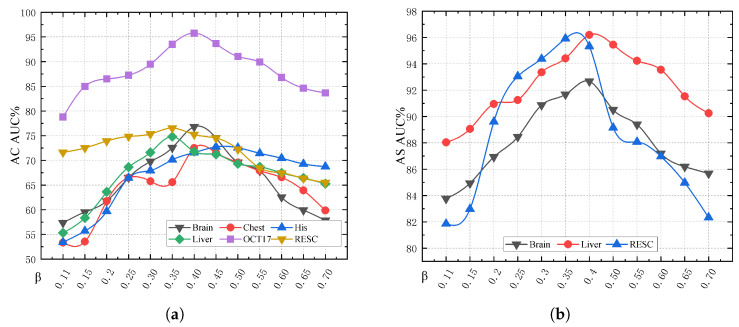
The figure shows that different constraint values have varying impacts on the results. Both anomaly classification (AC) and anomaly segmentation (AS) are similarly affected. (**a**) The figure demonstrates the impact of different constraint values on anomaly classification (AC) results. The optimal results are achieved when the constraint value is set to 0.4. (**b**) The results of anomaly segmentation (AS) are different for different constraint values. The results demonstrate the importance of irregular constraints for anomaly segmentation.

The given text discusses the concept of constraint rate β in models, specifically in the context of handling modal data. It states that different constraint rates reflect the model’s tolerance towards data within a particular mode. For image data, the constraint rate, represented by β, is crucial. The text finds that a constraint rate of 0.4 yields the best results for image tolerance in the model, as is shown in Figure 7. Furthermore, it suggests that constraining image data is particularly beneficial for enhancing the model’s ability to represent image features effectively. This insight indicates that adjusting the constraint rate can significantly impact the model’s performance, especially regarding image data.

### 3.3. Text Prompt

MedicalCLIP explores the relationship between the domain-generalization capabilities of the model and the types of data categories. For models pertaining to different data categories, we examined the variation of the model in relation to changes in data categories. Within the brain dataset, we conducted separate validations to assess the impact of varying numbers of categories and different types of object categories on the model’s performance.

To validate the impact of different text-generation methods on model performance, we conducted comparative tests for both category-dependent and category-independent text-generation. The results indicate that category-dependent textual descriptions enhance the model’s ability to guide anomaly-detection tasks. Conversely, category-independent text formulations exert a greater influence on the model’s domain-generalization capabilities.The results are shown in the Table 3.

**Figure 8:**
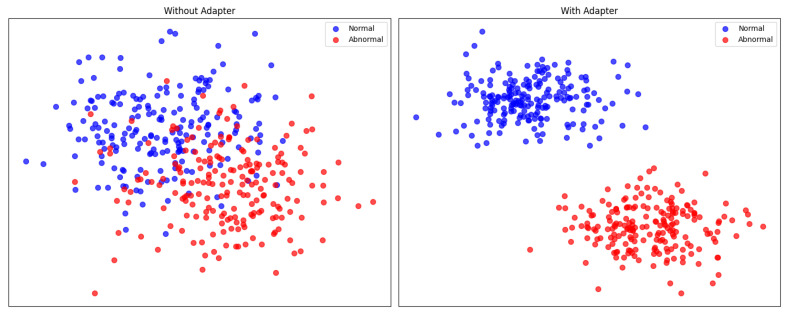
A fine-tuned adaptor layer allows filtering of features for anomaly-detection tasks. Filtering and constraints on image features can improve the model’s ability to discriminate between representations.

Feature Adaptation The initial CLIP model was primarily designed for classification tasks and has limited adaptability to anomaly-detection tasks. This paper refines the feature selection and adaptation through fine-tuning of image representations. The feature representation adaptive module employs a shallow network architecture for fine-tuning. As illustrated in Figure 8, after processing through the feature adaptive layer, the model is capable of delineating clearer boundaries. The results are shown in Table 4.

## 4. Discussion

Limited by the CLIP model’s understanding of semantic objects, it demonstrates weaker performance in anomaly-detection tasks. Through the customization of text prompts for anomaly detection, WinCLIP shows improved results. WinCLIP boosts performance using customized text prompts that are manually set, with their effectiveness critically dependent on the thoroughness of their text prompt. The textual information representation learned by CoOp relies more on training data, which, to some extent, limits the model’s generalization ability for unknown data. To make CLIP lean more towards semantic representation and enhance the model’s performance in segmentation tasks, a hierarchical and image block form of information representation is adopted. By using automatically generated text prompts, the restrictions of text prompts on visual representation are reduced, therefore improving generalization capability. Furthermore, to capture the details of image data, this paper establishes an asymmetric constraint-based intermodal contrast method. MedicalCLIP can perform fine-grained anomaly segmentation on different types of medical data, showcasing its ability to handle detailed information. This provides a new perspective for the application of the CLIP model in medical image anomaly-detection tasks.

## 5. Conclusions

In conclusion, this paper introduces an image–text irregular constraint method applied to medical image anomaly detection to achieve the ability to generalize the anomaly detection of different categories of data. A more professional and comprehensive description of textual information is established by generating textual hints through GPT. In this paper, modal content constraints are applied to text and images, and hierarchical information representation of image information is used to achieve more fine-grained textual semantic guidance and obtain more detailed anomaly-detection information. Combined with the multimodal contrast learning strategy, the method can be flexibly generalized to different types of data. This method provides new research ideas for anomaly detection of different categories of data due to existing methods in domain-generalization anomaly detection.

## Figures and Tables

**Table 1:** Compared to traditional anomaly-detection methods that use a single class and a single model, the method proposed in this paper has extremely strong domain-generalization capability. ✓ indicates possessing the corresponding capability.

Methods	Anomaly Score	Anomaly Segmentation	Model Unification	Domain Generalization
Traditional methods	✓	✓		
Few-shot methods	✓	✓		
Lvlms	✓	✓	✓	
Ours	✓	✓	✓	✓

**Table 2:** In domain generalization, different methods are compared. The main criteria for evaluation are the AUC for anomaly classification and anomaly segmentation. Bold indicates the best result, and underline indicates the second-best result.

Metrics	Methods	Brain	Chest	Histo Pathology	Liver	Retina OCT2017	Retina RESC	Metrics	Brain	Liver	Retina RESC
AC AUROC	WinCLIP [41]	66.49	70.86	69.85	64.20	46.64	42.51	AS AUROC	85.99	**96.20**	80.56
	April-GAN [47]	76.43	57.49	72.36	70.57	**92.61**	75.67		91.79	97.05	85.23
	CLIP [45]	55.63	60.62	61.87	55.78	40.42	40.46		80.11	82.35	76.46
	+ Prompt ens. [45]	55.95	61.45	62.53	58.62	41.78	41.32		91.26	89.86	79.65
	CoOp [21]	73.26	65.83	71.09	65.89	68.93	66.54		90.53	88.56	77.85
	Ours	**78.61**	**72.51**	**72.73**	**71.79**	85.79	**76.54**		**92.67**	95.63	**86.33**

**Table 3:** The impact of different types of text generation on model outcomes. Compared to category-specific generated text, category-independent text effectively enhances the model’s generalization ability for anomaly-detection tasks. Overall comparisons show that text generation has a greater impact on classification tasks than on segmentation tasks. Red indicates ✕ that the relevant description was not used, green indicates ✓ that it was used.

Textpormpt		AC AUROC						AS AUROC		
spd	cad	Brain	Chest	Histo Pathology	Liver	Retina 2017	Retina RESC	Brain	Liver	Retina RESC
✓	✕	76.45	68.28	68.75	68.93	81.98	72.93	89.34	92.53	83.49
✕	✓	77.56	69.93	69.91	71.36	83.47	74.84	91.45	93.56	85.28
✓	✓	78.61	72.51	72.73	71.79	85.79	76.54	92.67	95.63	86.33

**Table 4:** The effect of whether or not fine-tuning is performed on the model for which the image was acquired. The fine-tuned image representations are more suitable for anomaly-detection tasks. A more clearly differentiated representation of normal and abnormal can be created. AC denotes the anomaly classification indicator, and AS denotes the anomaly segmentation indicator.

Anomaly Classification	Withadaptor		Noadaptor	
Class	AC	AS	AC	AS
Brain	78.61	92.67	75.38	88.46
Chest	72.51	-	69.26	-
Histopathology	72.73	-	66.17	-
Liver	71.79	95.63	59.36	84.4
RetinaOCT2017	85.79	-	78.09	-
RetinaRESC	76.54	86.33	68.58	82.53

## Data Availability

Data are contained within the article.
